# Spatial association patterns between post-acute care services and acute care facilities in the United States

**DOI:** 10.1371/journal.pone.0240624

**Published:** 2020-10-12

**Authors:** Panayiotis D. Ziakas, Eleftherios Mylonakis

**Affiliations:** Warren Alpert Medical School of Brown University, Providence, RI, United States of America; The University of Aberdeen, UNITED KINGDOM

## Abstract

**Background:**

There is increasing demand for post-acute care services, which is amplified by the COVID-19 pandemic.

**Aims:**

We studied the pattern of spatial association between post-acute care services and acute care facilities and evaluated how geographic variability could influence their use.

**Methods:**

We compiled data on CMS-certified acute care and critical access hospitals and post-acute health care services (nursing homes, home health care services, inpatient rehabilitation facilities, long-term care hospitals, and hospice facilities). We used the colocation quotient (CLQ) to measure the magnitude and direction of association (clustering or segregation) between post-acute care providers and hospitals. This metric allows pairwise comparison of categorical data; a value <1 indicates spatial segregation and a value >1 spatial clustering. Unity marks the lack of spatial dependence (random distribution).

**Results:**

With the exception of nursing homes (CLQ 1.26), all other types of post-acute care providers are spatially segregated from rural critical access hospitals. Long-term care facilities ranked first (had the lowest global CLQ, 0.06), hospice facilities ranked last (had the highest global CLQ estimate, 0.54). Instead, post-acute care services either clustered with (inpatient rehabilitation 2.76, long-term care 2.10, nursing homes 1.37) or were only weakly segregated (home health care 0.86) from acute care hospitals. Home health care (1.44), hospice services (1.46), and nursing homes (1.08) spatially clustered with the same category of services. Results were robust in the sensitivity analysis and we provided illustrative examples of local variation for the states of MA and IA.

**Conclusion:**

Post-acute care services are isolated from critical access hospitals, and have a clustering pattern with the same category services and acute care hospitals. Such misdistribution of resources may result in both underuse and a substitution effect on the type of post-acute care between rural and urban areas and undermine public health during increasing demand, such as the COVID-19 pandemic.

## Introduction

After hospital discharge, many patients require additional support to recover, rehabilitate or manage chronic diseases. In 2013, 22.3% of all hospital discharges were to post-acute care services, with 50% discharged to home health care services and 40% to skilled nursing facilities [1]. Between 2000–2015, the use of post-acute services by Medicare beneficiaries has risen from 21% to 26%, while discharges home equally declined [2]. Medicare's fee-for-service expenditures were $ 58.9 billion for post-acute care in 2017 [3].

During the current COVID-19 pandemic, the increasing number and delayed recovery of hospitalized patients with COVID-19 [4], will further increase pressure on healthcare system and have a collateral effect on post-acute care services. The health insurance community, through “America's Health Insurance Plans,” has advocated in late March 2020 that patients negative for COVID-19 should be discharged to alternate post-acute care facilities, so vital nosocomial structures and resources are preserved to handle the crisis [5]. With the increasing demand for post-acute care, fueled in the COVID-19 era, we explored whether a pattern of association exists between different provider types and hospital facilities and how such pattern could affect their use.

## Methods

### Sources

We compiled publicly available data from the Centers for Medicare & Medicaid Services (CMS), available on the official Medicare Websites and Directories (https://cms.gov and https://data.medicare.gov). Hospital location data were extracted by the hospital compare datasets and were used to construct the geospatial network that served as the reference core for our main analysis. Inpatient rehabilitation facilities, long-term care hospitals, nursing homes, hospice facilities, and home healthcare providers served for comparisons.

### Metrics

We used the colocation quotient (CLQ) to measure the spatial dependence between categories of interest in the given population of CMS-certified facilities. Specifically, for two categories of A and B points, CLQ_A→B_ denotes the spatial dependence of type A points to B points, that is how B points attract type-A points. It is defined as the ratio of expected proportions of B's among A's nearest neighbors. A value >1 indicates attraction of A points to B points (more B neighboring points than expected), whereas a value <1 indicates segregation of type A points from type B (fewer neighboring B points than expected). Unity denotes absence of spatial dependence (random distribution).

Spatial dependence is not reciprocal but asymmetric for different categories, that is A to B point attraction is not expected to be the same as B to A attraction unless we estimate the same category (e.g. A to A points) spatial dependence [6]. CLQ uses distance ranks instead of actual distance metrics to provide a pairwise comparison of categorical subsets. The methodology expands to geographically weighted (using spatial neighborhoods) global and local CLQ estimates. Monte Carlo simulation permits significance testing [7]. While a global CLQ provides an overall description of the spatial pattern between two categories, local CLQs describe spatial variability between point sets [8,9].

The selection of bandwidth is a critical element for analysis [10], and is performed in advance through a preliminary exploration of the actual data. A fixed radius (bandwidth) draws the area around a point within which colocating points should lie. Instead, if an adaptive filter is chosen, the bandwidth can vary from point-to-point to secure a minimum number of colocating points in the neighborhood. The latter approach is warranted when spatial points are unevenly distributed across the region to study and adds to reliability and validity of results [6,9,11,12]. For each type of filter, different kernel density functions can then be used to establish the geographical weights associated with all potential colocating points.

The kernel density function used here is the box function (gives an equal weight of 1 to the closest neighbors) for global estimates. There are also distance-based functions (the farther a neighbor locates from a facility, the less weight it is assigned). A Gaussian kernel was used for local estimates [8,9]. The choice of kernel weighting will have minimal impact on results, as opposed to bandwidth selection which has a substantial impact. In the absence of a steadfast rule to optimize bandwidth selection, an “ideal” bandwidth should reflect the actual distances between the spatial points. Empirically, a large bandwidth will smooth the density surface attenuating local detail, whereas a small bandwidth will highlight local differences at the expense of losing important aggregation patterns [11,13].

We geocoded facility locations using Stata (College Station, TX, USA) and OpenCage Data’s geocoding application programming interface [14]. For computing measures of association, we used the free tools by Kronenfeld [15] and Wang [9]. For data visualization, we used the GeoDa open-source tool for geographic data analysis. We used arc distances for analysis to compensate for the curvature of the earth since our location data are in the longitude-latitude form (not projected) [11]. Institutional approval was not required, as the study does not involve human subjects and compiles publicly available data.

## Results

As of February 2020, there was compiled information on 37,557 healthcare providers of interest across 50 states and District Columbia (not considering Puerto Rico and outlying US territories). A total of 37,400 were located across the contiguous US area (not including Hawaii and Alaska) and were used for analysis (Table 1).

**Table 1:** Nationwide provider data type and services, for contiguous US area (Alaska & Hawaii excluded from analysis).

Type of provider	N	Type of service
**Acute care hospital**	3,204	Short-term, inpatient medical care for surgery, acute medical condition or illness
**Critical access hospital**	1,332	Rural hospitals, providing essential services in rural communities
**Home health care Agency**	11,131	Health services at home, may include skilled nursing care, medical care, physical therapy, care by home health aides etc.
**Hospice**	4,817	Care for the terminally ill
**Inpatient rehabilitation facility**	1,159	Rehabilitation hospitals and rehabilitation units in hospitals to provide intensive rehabilitation programs
**Long-term care hospital**	375	Extended medical and rehabilitative care (>25 days) for complex and chronic conditions
**Nursing home**	15,382	Skilled nursing care, rehabilitation services for disabled and sick persons

Compiled from cms.gov & data.medicare.gov in February 2020; Puerto Rico and outlying US territories not considered; children’s, psychiatric hospitals and acute care, U.S. Department of Defense hospitals not included.

We based bandwidth selection on the distribution patterns of hospital facilities, including 3,204 acute care hospitals and 1,332 critical access hospitals. According to Pew Research [16], rural Americans live an average of 10.5 miles from the nearest hospital, compared with 5.6 miles for people in suburban areas and 4.4 for those in urban areas, with more than 80% living within a 10-mile radius from the nearest hospital (using arc distances to account for the curvature of the Earth). Therefore, selecting a 10-mile distance bandwidth provides a wide population coverage and gives an average of 4 neighboring acute care facilities. As such, we opted for an adaptive bandwidth of k = 4 neighbors as the reference core for our main analysis.

We selected an adaptive bandwidth that uses distance ranks (that is the first closest neighbor, second closest neighbor, etc.) instead of using actual metric distances, to compensate for unevenly distributed data. This applies specifically to critical care hospitals in rural areas, 90% of which do not have a neighboring facility within the 10-mile bandwidth. For sensitivity analysis, we chose k = 1 as the most conservative scenario (to account for the paucity of hospital facilities in rural areas). On the other hand, selecting a network of k = 17 nearest neighbors (corresponding to the cubic root of observations) is an approximation recommended to ensure equal or near equal spatial coverage [11,17], but a less conservative approach, that would attenuate local geospatial patterns and smooth estimates. As such, adaptive bandwidths of k = 1 and k = 17 were used to test the stability of our main analysis. Of note, as we opted for an adaptive bandwidth of 4 nearest neighbors for our main analysis of global CLQs (by category), we did not calculate local CLQs (per facility) at the nationwide level, because they become underpowered for small counts and are not recommended if there are fewer than ten observations for calculation [6].

We divided CLQ estimates in four descriptive classes for easy interpretation: <0.50 for moderate-to-strong spatial dispersion (segregation); 0.50–1.00 for weak-to-moderate spatial dispersion (segregation); 1.01–1.50 for weak-to-moderate colocation (clustering) and >1.50 for moderate to strong colocation (clustering) [8] (Table 2).

**Table 2:** Colocation quotients between types of providers and hospital facilities. Main analysis for an adaptive bandwidth of k = 4 nearest neighbors. Sensitivity analysis for k = 1 (the most conservative) and k = 17 (the least conservative scenario).

Type of provider	Acute care hospitals			Critical access hospitals			Same type		
	k = 1	**k = 4**	k = 17	k = 1	**k = 4**	k = 17	k = 1	**k = 4**	k = 17
**Home Health Care**	0.73	**0.86**	0.83	0.51	**0.45**	0.46	1.43	**1.44**	1.41
**Hospice**	0.65	0.97*	0.94	0.45	**0.54**	0.60	1.34	**1.46**	1.48
**Inpatient rehabilitation**	6.85	**2.76**	1.39	0.18	**0.12**	0.35	0.31	**0.65**	0.94*
**LTCH**	2.92	**2.10**	1.32	0.15	**0.06**	0.12	0.22*	1.23*	1.14*
**Nursing home**	1.48	**1.37**	1.13	2.01	**1.26**	1.01*	1.09	**1.08**	1.10

* non significant estimates through in Monte-Carlo simulation; all remaining estimates significant with p-values <0.001.

Four types of facilities (hospice, home health care, inpatient rehabilitation, and long-term care) are distributed in moderate-to-strong isolation from the critical access hospitals across the US. Long-term care facilities were the most segregated category (CLQ 0.06) and hospice facilities the least segregated (CLQ 0.54) from critical access hospitals. Estimates remained within the same descriptive class in sensitivity analyses, thus segregation patterns did not change. Nursing homes was the only category to show weak-to-moderate spatial clustering with critical access hospitals (CLQ 1.26). However, the spatial pattern become strong clustering (CLQ 2.01) for k = 1 (the most conservative scenario) and random association (not different from unity) for k = 17 (the least conservative scenario).

The spatial associations between post-acute services and acute care hospitals showed different pattern: Specifically, long-term care facilities and inpatient rehabilitation services had global CLQs higher than two (2.10 and 2.76, respectively) indicating strong spatial clustering with acute care hospitals. These patterns held with increasing magnitude (CLQs 2.92 and 6.85, respectively) for k = 1 and survived as weak-to-moderate clustering (CLQs 1.32 and 1.39, respectively) in the least conservative scenario (k = 17) of sensitivity analysis. Spatial analysis of nursing homes demonstrated weak-to-moderate clustering (CLQ 1.37) with acute care hospitals, an association that held in sensitivity analyses. This is opposed to home healthcare agencies that appeared weekly isolated (spatially segregated) from acute care hospitals, with a global CLQ of 0.86. The pattern remained unchanged in the sensitivity analysis. Analysis of hospice services lacked spatial dependence with acute care hospitals in the main analysis, which changed to weak-to-moderate segregation for k = 1 (CLQ 0.65) and k = 17 (CLQ 0.94) nearest neighbors in the sensitivity analysis.

Post-acute care services tended to cluster with the same category services. Analysis of home health care facilities (CLQ 1.44), hospice services (CLQ 1.46), and nursing homes (CLQ 1.08) demonstrated weak-to-strong clustering with the same category facilities. These associations remained robust in the sensitivity analysis. Hospice facilities had weak-to-moderate segregation with themselves (CLQ 0.65), which became more prominent in the most conservative scenario (CLQ 0.31 for k = 1) and vanished in the least conservative scenario (CLQ not different from unity for k = 17). Long-term care facilities lacked an association pattern with themselves (CLQ not different from unity).

We selected two states as representative examples to illustrate local associations between post-acute care services and hospitals: Massachusetts (MA), is the New England’s hub for hospital services; and Iowa a rural area with the largest difference between critical access to acute care hospitals (CMS data). At the state level, we used an adaptive bandwidth of near equal coverage for land facilities and a Gaussian kernel.

In MA, for an adaptive bandwidth of near-equal coverage (n = 10) and a Gaussian kernel, the majority of post-acute land facilities (74.0%) had a weak-to-moderate spatial dependence with acute care hospitals, either a clustering (32.9%) or a segregation (41.1%) pattern. 12.5% had a moderate-to-strong clustering with acute care hospitals, a pattern mainly seen in Boston area. Also, 13.5% had moderate-to-strong segregation with acute care hospitals.

In IA, for an adaptive bandwidth of near-equal coverage (n = 10) and a Gaussian kernel, the majority of post-acute land facilities (59.4%) have a spatial segregation with critical access hospitals, either a moderate-to-strong (42.1%) or a weak-to-moderate segregation (17.3%) pattern. A moderate-to-strong segregation pattern with critical access hospitals occurred because post-acute land facilities preferentially concentrate near IA cities of Des Moines, Davenport, Cedar Rapids and Waterloo. The remaining 40.6% of post-acute care facilities had, either a weak-to-moderate (21.3%) clustering or a moderate-to-strong clustering (19.3%) with critical access hospitals.

## Discussion

We explored the nationwide distribution between post-acute care services and hospitals. With the exception of nursing homes, all types of post-acute services were spatially segregated from critical care hospitals. Specifically, inpatient rehabilitation facilities and long-term care facilities strongly clustered with acute care hospitals, whereas home health care services had weak spatial segregation from acute care hospitals. Nursing homes was the only category to show moderate clustering with both critical access and acute care hospitals, while, home health care, hospice services, and nursing homes had spatial clustering with the same category facilities.

The use of post-acute care services after hospital discharge is a fast-growing area in the United States [18,19] and geographic disparities may account for differences in healthcare spending [20,21]. The location of hospitals and post-acute care facilities may moderate the use of services. Lower population density and the relative lack of specialized care, make critical care hospitals less suitable for post-acute care, including the long-term care hospitals which were strongly segregated from critical access hospitals. The spatial segregation between post-acute care facilities and critical care hospitals in rural areas could explain why post-acute care use was less frequent after orthopedic procedures (hip fracture and hip replacement surgeries) in critical care hospitals [22,23].

Spatial dependence may also influence the type of services utilized. Home health care services and inpatient rehabilitation facilities were spatially segregated from rural hospitals whereas nursing homes had weak-to-moderate clustering. As expected, rural patients were less likely to be discharged to home health care than skilled nursing facilities [23,24] or an inpatient rehabilitation facility [24]. Differences in premorbid conditions and the lack of a clear consensus on the patients who need post-acute care or what kind of care is warranted after discharge can arguably drive these disparities [25,26].

However, the availability and distance of post-acute services is the major driver of their use and selection and, if there are fewer inpatient rehabilitation services in an area than nursing homes then more discharges are referred to a nursing home. The farther away an inpatient rehabilitation service locates, the less likely is for a patient to use after discharge and refer instead to a nursing home [27]. As a result, spatial clustering or segregation between post-acute care services and hospitals can drive both a quantitative difference in use and a substitution effect (on what type of service to use) as well. Rural areas may be regarded as less attractive options to invest in post-acute care, given the low population density and the high fixed costs to operate with lower margins [3], which undermine the financial viability of such projects.

The spatial isolation between post-acute care services and critical access hospital contrasts to the spatial dependence between post-acute care facilities and acute care hospitals. The local estimates vary from strong clustering (seen for inpatient rehabilitation and long-term care hospitals) to only weak segregation (for home health care services). Three-quarters of inpatient rehabilitation services locate as distinct units in acute care hospitals and a quarter are free-standing providers, which is a plausible explanation for their strong clustering [3]. Long-term care hospitals treat the patients who need extended high-level care, after discharge from acute care hospitals. Nursing homes, the most populated post-acute provider category with more than 15,000 facilities nationwide, has only 4% of facilities located in a hospital (by CMS database). Up to half of the hospital discharges for post-acute care end up in a nursing home [1]; vice versa, early and late readmissions to hospitals from nursing homes are frequent and may sum up to approximately 30% [28–30]. Hospice offers palliative services and care for the terminally ill with approximately 51% of Medicare decedents using it (and a total of 1.5 million users) in 2018 [31], and hospice-to-hospital transitions are limited [32,33], while home health care is a consumer-oriented service delivered at home rather than a hospital-oriented activity.

Our study provides useful insights into the current spatial associations between different levels of health care delivery. These associations are not random but related to a background of geographical and economic diversity. Noteworthy, these patterns of association were longitudinally shaped in the absence of a major epidemic, and may become of critical importance during and after the COVID-19 era. COVID-19 has devastated post-acute care, adding cases and survivors who experience a spectrum of clinical, functional, and psychological sequelae [34]. Recent data indicate that after hospitalization, less than 5% of survivors will require inpatient rehabilitation, as opposed to 45% of survivors who will require post-acute support [35,36]. There is early evidence to indicate geographical variation in post-acute response to COVID-19 surge. For example, a survey found that more home health care agencies in urban areas had cared for confirmed (31%) and recovered (24%) COVID-19 patients than in rural areas (18% and 6%, respectively). Moreover, urban health care agencies had confronted more staffing challenges, protective equipment shortages and decrease in patient census (and revenues) compared to rural facilities [37]. There is also absence of a uniform response across states and local providers. For example, some local agencies completely ban the transfer of patients to post-acute services regardless of COVID-19 status, while others accept all patients to relieve hospitals from overwhelming capacity constraints [38]. Post-acute care is expected to have a paramount importance in COVID-19 era and geography will be a local moderator. For example, in urban areas, post-acute services will have to serve as valve to preserve acute care hospital capacity or re-purposed as designated post-COVID-19 centers. These roles may not apply in the rural setting and the optimal nationwide and regional resource use for post-acute care during the epidemic is a matter of ongoing debate [38].

In conclusion, post-acute care services in the United States are spatially isolated from critical access hospitals, which should be considered a disparity with potential impact on their use. Post-acute-care facilities aggregate around acute care hospitals, with a spatial pattern related to the type of service they provide. The surge for increased hospital care services during an epidemic such as COVID-19, is also expected to push post-acute services to their limits, with a disproportionate impact in regions where such services are spatially segregated from hospital providers. Underuse of post-acute care in these areas would adversely affect population health.

## Supporting information

S1 Dataset Memo: IRF = Inpatient Rehabilitation Facility; NH = Nursing Home; hh_care = Home Health Care; acute_care = Acute Care Hospital; crit_care = Critical Access Hospital; hospice = Hospice; LTCH = Long-Term Care Hospital; g_lon = longitude; g_lat = latitude. (TXT)
